# Comparison of brain magnetic resonance imaging between myotonic dystrophy type 1 and cerebral autosomal dominant arteriopathy with subcortical infarcts and leukoencephalopathy

**DOI:** 10.1371/journal.pone.0208620

**Published:** 2018-12-06

**Authors:** Hyunjin Kim, Young-Min Lim, Yeo Jin Oh, Eun-Jae Lee, Kwang-Kuk Kim

**Affiliations:** Department of Neurology, Asan Medical Center, University of Ulsan College of Medicine, Seoul, Republic of Korea; McLean Hospital, UNITED STATES

## Abstract

**Background:**

Anterior temporal lobe hyperintensities detected by brain MRI are a recognized imaging hallmark of cerebral autosomal dominant arteriopathy with subcortical infarcts and leukoencephalopathy (CADASIL). Because similar findings may be present in patients with myotonic dystrophy type 1 (DM1), the brain MRI in these two diseases is often misinterpreted. We compared the MRI findings between the two entities to examine whether they display distinctive characteristics.

**Methods:**

This retrospective, cross-sectional study reviewed medical records of patients with DM1 or CADASIL admitted to Asan Medical Center between September 1999 and September 2017. We compared the frequency and grades of white matter changes in specific spatial regions between the groups according to age-related white matter change scores. We also evaluated the presence of cerebral microbleeds.

**Results:**

A total of 29 patients with DM1 and 68 with CADASIL who had undergone MRI were included in the analysis. The overall prevalence of white matter hyperintensities was 20 (69%) and 66 (97%) in DM1 and CADASIL, respectively (*p* < 0.001), whereas the frequency of anterior temporal lobe hyperintensities was comparable between the groups (10 [34.5%] in DM1 vs. 35 [51.5%] in CADASIL, *p* = 0.125). The brain MRI of patients with DM1 revealed more limited involvement of the frontal, parieto-occipital, external capsule and basal ganglia regions compared with imaging in patients with CADASIL. Cerebral microbleeds were not observed in any case of DM1 but were present in 31 of 45 (68.9%) cases of CADASIL.

**Conclusions:**

Anterior temporal lobe involvement in DM1 is not infrequent compared with CADASIL. However, because brain MRI in patients with DM1 lacks other distinctive features seen in CADASIL, imaging might assist in differentiating these two conditions.

## Introduction

Hyperintense white matter lesions in the anterior temporal lobe detected by brain magnetic resonance imaging (MRI) could be attributed to a number of conditions. However, they are a recognized imaging hallmark of cerebral autosomal dominant arteriopathy with subcortical infarcts and leukoencephalopathy (CADASIL) [1]. The use of MRI to determine involvement of the anterior temporal lobe and external capsule is reported highly sensitive and specific for differentiating CADASIL from sporadic leukoaraiosis [2].

Myotonic dystrophy type 1 (DM1) is caused by a CTG repeat expansion in the 3’ untranslated regions of *DMPK* on chromosome 19 and is characterized by progressive myopathy, myotonia and multi-organ involvement [3]. Although brain involvement in DM1 has been demonstrated in many neuroimaging and pathology studies, the characteristic MRI patterns in DM1 are relatively less recognized. Additionally, anterior temporal lobe hyperintensities resembling those of CADASIL can be seen in patients with DM1. The imaging in these two diseases could therefore be misinterpreted [4, 5].

When evaluating patients exhibiting anterior temporal lobe MRI hyperintensities but who do not have clinical manifestations indicative of a particular diagnosis, clinicians have to rely mainly on the characteristics seen on MRI. In the present study, we aimed to identify distinctive brain MRI characteristics in patients with DM1 or CADASIL to determine whether the imaging could aid in successfully differentiating the two diseases.

## Materials and methods

### Patients

In this retrospective, cross-sectional study, we reviewed the medical records of 149 patients with DM1 and 80 with CADASIL diagnosed by genetic analysis at Asan Medical Center (Seoul, South Korea) between September 1999 and September 2017. Since leukoaraiosis worsens with age, only patients aged 18-to-65 years for whom brain MRI scans were available were included. The review comprised available clinical and laboratory data, including demographics, stroke risk factors (previously diagnosed and treated hypertension or systolic/diastolic blood pressure ≥140/90 mmHg, previously diagnosed and treated diabetes mellitus or a hemoglobin A1c ≥6.5% and current smoking), and the results of genetic analysis.

### Genetic analysis

#### DM1

Genomic DNA was isolated from peripheral blood using a Puregene DNA isolation kit (Gentra Systems, Minneapolis, MN, USA). To analyze CTG expansion of *DMPK*, polymerase chain reaction (PCR) was performed using primers the 5′-CAGTTCACAACCGCTCCGAGC-3′ and 5′-CGTGGAGGATGGAACACGGAC-3′. Subsequently, the PCR fragments were subjected to gel electrophoresis, capillary transfer and hybridization with a biotin-labelled (CAG)_10_ probe. Southern blotting was performed using a DNA Detector Southern blotting kit (KPL, Gaithersburg, MD, USA).

#### CADASIL

Genomic DNA was isolated from peripheral blood using a Puregene isolation kit (Gentra). Eleven exons (2–11 and 18) of *NOTCH3* and their flanking intron sequences were amplified using PCR with 11 primer sets. Following amplification, the PCR products were separated by electrophoresis on 1.2% agarose gels in the presence of ethidium bromide to verify their size and purity. Subsequent DNA sequencing and analysis were performed on an ABI 3130XL Genetic Analyzer (Applied Biosystems, Foster City, CA, USA) using a BigDye Terminator v3.1 Cycle Sequencing Ready Reaction kit (Applied Biosystems, Foster City, CA, USA) according to the manufacturer’s instructions. The sequencing primers were the same as those for PCR.

### Brain MRI

Brain MRI was performed with a 1.5-T (Magnetom Avanto, Siemens Medical Solutions, Malvern, PA, USA) or a 3.0-T (Philips Achieva, Philips Medical Systems, Andover, MA, USA) MRI system. Axial T2, fluid attenuation inversion recovery (FLAIR), and gradient-echo T2*-weighted (GRE) images were obtained. The FLAIR images were acquired from a fast spin-echo sequence with the following parameters: repetition time/echo time, 9000/100 ms; inversion time, 2500 ms; and matrix, 256 × 224. The GRE images included the following parameters: slice thickness, 5 mm; inter-slice gap, 2 mm; number of axial slices, 20; field of view, 250 mm; repetition time, 400 ms; echo time, 30 ms; flip angle, 20°; and matrix, 256 × 192. White matter abnormalities were graded in T2 and FLAIR images according to the age-related white matter change (ARWMC) scoring system [6] as follows: 0, absent; 1, focal; 2, initially confluent; and 3, diffuse involvement for any lesions in the frontal, parieto-occipital, infratentorial, anterior temporal, or external capsule regions. For lesions in the basal ganglia, ARWMC scoring was 0, absent; 1, one focal lesion >5 mm; 2, >1 lesion; and 3, confluent. Only the left hemispheres were scored. An ARWMC score ≥1 was defined as the presence of a white matter change. Cerebral microbleeds were defined according to the criteria provided by Greenberg *et al*. [7]. The presence of cerebral microbleeds was verified using the GRE images. The ARWMC scores and the presence of cerebral microbleeds were determined by consensus between two investigators (H. Kim and Y.J. Oh) who were blinded to the clinical data. A third investigator (E.-J. Lee) was consulted in cases of disagreement.

### Statistical analysis

Descriptive data are presented as frequencies and percentages for categorical variables and mean ± standard deviation for continuous variables. A *t*-test was performed to compare normally distributed variables. The chi-square test or Fisher’s exact test was used for two-group comparisons of categorical variables as appropriate. Ordinal logistic regression analysis with adjustment for covariates (age, hypertension, diabetes and smoking) was performed to evaluate the relationship between disease diagnosis and ARWMC score. Two-sided *p* values <0.05 were considered to indicate statistical significance. Receiver operating characteristic (ROC) curve analysis of the ARWMC scores was performed. The thresholds for distinguishing the two diseases were calculated, and the sensitivity and specificity of these thresholds were tested. All statistical analyses were performed with IBM SPSS Statistics for Windows version 21.0 (SPSS, Armonk, NY, USA).

### Ethics statement

The institutional review board of Asan Medical Center approved this study (2017–0773) and waived the need for informed consent based on the retrospective study design.

## Results

A total of 120 patients with DM1 were excluded because MRI had not been performed. There were no significant differences in characteristics (age, sex, CTG repeat number, electrocardiography and stroke risk factors) between patients with DM1 who did or did not undergo MRI (S1 Table). Twelve CADASIL patients were excluded because of age (<18 or >65 years). Thus, 29 patients with DM1 and 68 with CADASIL were included in the final analysis.

The characteristics of both groups are presented in Table 1. The mean age at onset (39.4 ± 13.6 in DM1 vs. 46.9 ± 9.4 in CADASIL, *p* = 0.002) and at MRI assessment (44.4 ± 13.1 in DM1 vs. 50.3 ± 10.0 in CADASIL, *p* = 0.036) was lower in the DM1 group. About half the patients in each group were men. Among patients with DM1, the main symptom at onset was gait disturbance (41.4%), followed by hand weakness/stiffness (37.9%) and dysarthria/dysphagia (20.7%). In those with CADASIL, the main presenting symptom was hemiparesis (25.0%), followed by headache (23.5%) and dysarthria/dysphagia (11.8%). The prevalence of stroke risk factors was comparable between the groups.

**Table 1:** Characteristics of patients with DM1 or CADASIL.

	DM1 (n = 29)	CADASIL (n = 68)	*p* value
Age at onset (years)	39.4 ± 13.6	46.9 ± 9.4	0.002
Age at MRI (years)	44.4 ± 13.1	50.3 ± 10.0	0.036
Male	17 (58.6)	33 (48.5)	0.363
Symptoms at onset			< 0.001
Hand weakness/stiffness	11 (37.9)	2 (2.9)	
Gait disturbance	12 (41.4)	3 (4.4)	
Dysarthria/dysphagia	6 (20.7)	8 (11.8)	
Sensory disturbance	0	9 (13.2)	
Headache	0	16 (23.5)	
Dizziness	0	6 (8.8)	
Hemiparesis	0	17 (25.0)	
Depression	0	2 (2.9)	
Cognitive impairment	0	5 (7.4)	
Stroke risk factors			
Hypertension	2 (6.9)	13 (19.1)	0.218
Diabetes mellitus	5 (17.2)	4 (5.9)	0.122
Smoking	5 (17.2)	25 (36.8)	0.057
CTG repeats	315.5 ± 175.4	-	-
*NOTCH3* mutations involving cysteine	-	47 (69.1)	-

Values are presented as mean ± standard deviation or n (%).

CADASIL, cerebral autosomal dominant arteriopathy with subcortical infarcts and leukoencephalopathy; DM1, myotonic dystrophy type 1 and MRI, magnetic resonance imaging

Table 2 presents the MRI characteristics in each group. Although white matter hyperintensities were observed in 20 (69%) patients with DM1 compared with 66 (97%) with CADASIL, the frequency of anterior temporal lobe hyperintensities was comparable between the groups (34.5% in DM1 vs. 51.5% in CADASIL, *p* = 0.125). The MRI evaluation revealed that involvement of the frontal, parieto-occipital lobes, external capsule and basal ganglia was less common in DM1 than in CADASIL. Patients with DM1 had significantly lower ARWMC scores in the frontal, parieto-occipital and external capsule regions compared with those in the CADASIL patients. In contrast, the ARWMC scores in the anterior temporal and infratentorial regions did not differ significantly between the two groups. The prevalence of stroke was 13.8% in DM1 and 77.9% in CADASIL. Cerebral microbleeds were not observed in any patient with DM1 but were present in 31 of 45 (68.9%) patients with CADASIL. The percentages of patients scanned by 1.5-T or 3.0-T MRI did not differ between the groups.

**Table 2:** Brain MRI characteristics in patients with DM1 or CADASIL.

	DM1 (n = 29)	CADASIL (n = 68)	*p* value
White matter changes			
Total	20 (69.0)	66 (97.1)	< 0.001
Frontal	11 (37.9)	64 (94.1)	< 0.001
Parieto-occipital	17 (58.6)	66 (97.1)	< 0.001
Anterior temporal	10 (34.5)	35 (51.5)	0.125
External capsule	3 (10.3)	55 (80.9)	< 0.001
Infratentorial	1 (3.4)	12 (17.6)	0.100
Basal ganglia	0 (0.0)	42 (61.8)	< 0.001
ARWMC scores			
Frontal	0.66 ± 0.94	2.29 ± 0.89	< 0.001^a^
Parieto-occipital	1.07 ± 1.00	2.37 ± 0.75	< 0.001^a^
Anterior temporal	0.72 ± 1.13	1.06 ± 1.18	0.205^a^
External capsule	0.14 ± 0.44	1.26 ± 0.87	< 0.001^a^
Infratentorial	0.04 ± 0.19	0.26 ± 0.61	0.110^a^
Basal ganglia	0.00 ± 0.00	1.06 ± 1.02	0.043^b^
Stroke	4 (13.8)	53 (77.9)	< 0.001
Presence of microbleeds	0/10 (0.0)	31/45 (68.9)	< 0.001
1.5-T MRI	15 (51.7)	44 (64.7)	0.230

Values are presented as mean ± standard deviation or n (%).

^a^Ordinal logistic regression analysis with adjustments for covariates including age, hypertension, diabetes mellitus and smoking.

^b^R Package ordinal logistic biplot with ridge estimators for logistic regression (Le Cessie S, Van Houwelingen JC. Ridge estimators in logistic regression. J R Statistical Society Ser C Appl Statistics. 1992;41(1):191–201).

ARWMC, age-related white matter change; CADASIL, cerebral autosomal dominant arteriopathy with subcortical infarcts and leukoencephalopathy and DM1, myotonic dystrophy type 1

**Fig 1:**
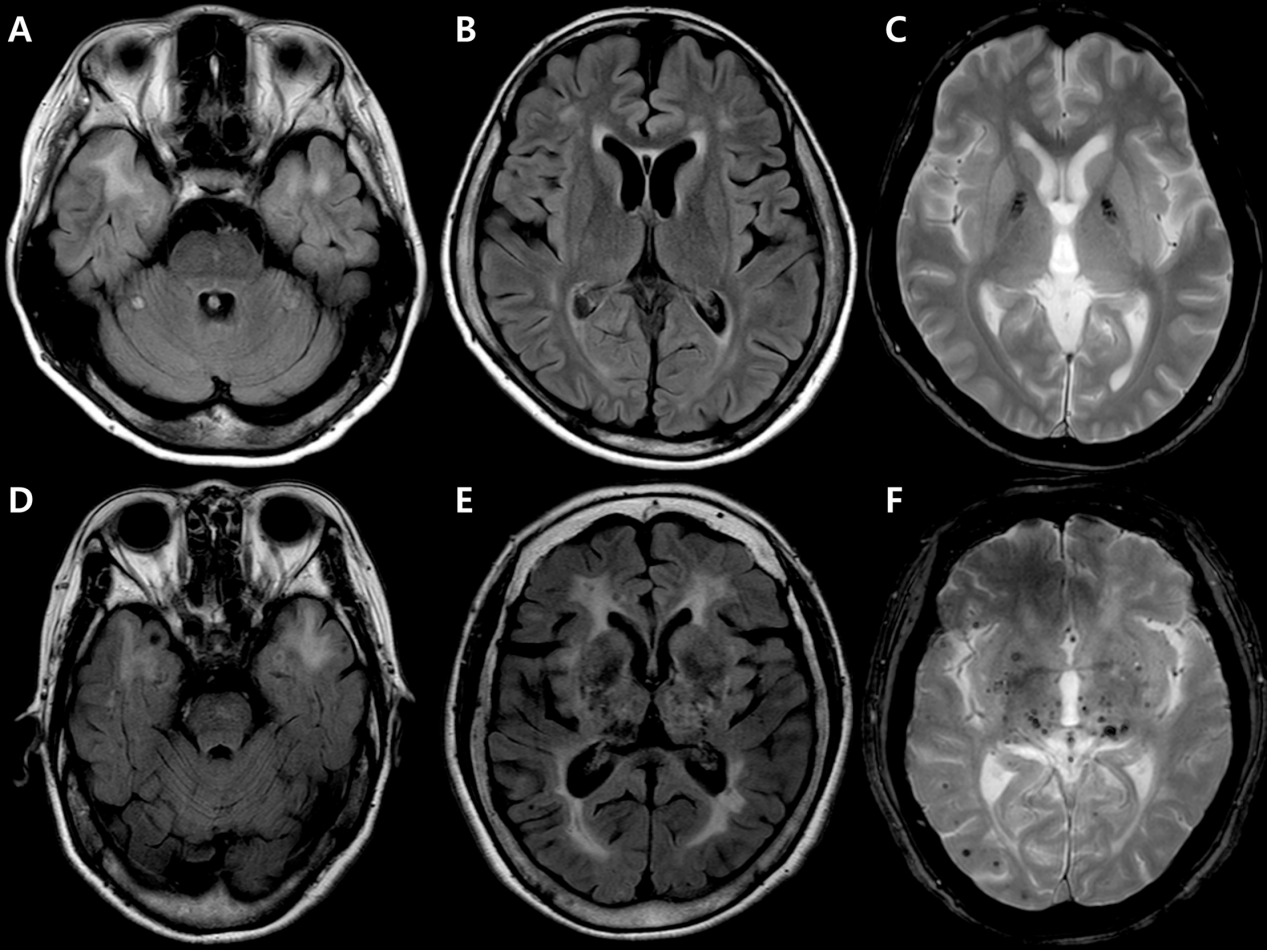
Representative MRI of patients with DM1 or CADASIL. Axial fluid attenuation inversion recovery (FLAIR) images showing bilateral anterior temporal hyperintensities in DM1 (**A**) and CADASIL (**D**). Axial FLAIR images showing more limited involvement of frontal, parieto-occipital, external capsule and basal ganglia regions in DM1 (**B**) compared with CADASIL (**E**). Axial gradient-echo T2*-weighted (GRE) images showing absence of microbleeds in DM1 (**C**) compared with multiple microbleeds observed in CADASIL (**F**).

The ROC curve analysis of the ARWMC scores (Table 3) revealed an area under the curve of a composite (frontal lobe + parieto-occipital lobe + external capsule + basal ganglia) score that was greater than any other individual score. A composite score ≥5 suggested a diagnosis of CADASIL. Using that score as the cut off yielded a sensitivity of 85% and specificity of 93% for CADASIL rather than DM1. Representative MRI images from patients with DM1 and CADASIL are presented in Fig 1.

**Table 3:** ROC analysis of ARWMC scores.

	AUC	95% CI	Threshold	Sensitivity (95% CI)	Specificity (95% CI)
Frontal	0.873	0.80–0.95	2	82.4 (71.6–89.6)	75.9 (57.9–87.8)
Parieto-occipital	0.834	0.75–0.92	2	89.7 (80.2–94.9)	55.2 (37.6–71.6)
Anterior temporal	0.582	0.46–0.71	1	51.5 (39.8–63.0)	65.5 (47.4–80.1)
External capsule	0.859	0.78–0.94	1	80.9 (70.0–88.5)	89.7 (73.6–96.4)
Infratentorial	0.573	0.45–0.69	1	17.6 (10.4–28.4)	96.6 (82.8–99.4)
Basal ganglia	0.809	0.73–0.89	1	61.8 (49.9–72.4)	100 (88.3–100)
Composite	0.925	0.87–0.98	5	85.3 (75.0–91.8)	93.1 (78.0–98.1)

ARWMC, age-related white matter change; AUC, area under the curve; CI, confidence interval and ROC, receiver operating characteristic

## Discussion

Following a study by O’Sullivan *et al*. in 2001 [2], anterior temporal lobe hyperintensities were considered a radiologic marker of CADASIL. However, anterior temporal lobe white matter lesions have also been observed in approximately one-third of patients DM1, as revealed in the present study and in a recent systematic review [8]. Our study established that the frequency of such lesions and the ARWMC scores in the anterior temporal lobe did not differ significantly between the DM1 and CADASIL groups. Both groups exhibited confluent white matter lesions in the temporopolar regions, such that the brain MRI findings of these two diseases could be radiologically confusing. In addition, the main symptoms at onset in patients with DM1 were gait disturbance, hand weakness and dysphagia/dysarthria, mimicking the symptoms of a stroke, which are also the main symptoms of the patient with CADASIL [9]. However, MRI exhibited specific features that could aid in discriminating DM1 from CADASIL. Firstly, the frontal and parieto-occipital lobes, external capsule and basal ganglia were involved to a lesser extent in DM1 than in CADASIL. In particular, the frequency of external capsule involvement, another reported imaging hallmark of CADASIL, was lower in patients with DM1. Secondly, cerebral microbleeds were not observed in any of the GRE images of patients with DM1, whereas 69% of those with CADASIL had microbleeds. This might therefore be another useful discriminator between these two diseases.

Even though the MRI in patients with DM1 and CADASIL revealed similar anterior temporal lobe hyperintensity patterns, the pathologic mechanisms underlying the two diseases are distinct. DM1 is caused by unstable expanded CTG repeats in the non-coding regions of *DMPK*, resulting in intranuclear accumulation of mutated transcripts (RNAopathy) and mis-splicing of numerous transcripts (spliceopathy) [10]. Neurofibrillary tangles found in the brains of patients with DM1 also distinguish DM1 as a tauopathy [11]. Tau mis-splicing in DM1 has been demonstrated at both the RNA and protein levels [12]. Thus, white matter changes on MRI in DM1 might be a manifestation of tau-related neurodegeneration. In contrast, CADASIL is caused by mutations in NOTCH3 which accumulate in small arteries [9]. Therefore, white matter hyperintensities on MRI of patients with CADASIL are a manifestation of chronic small artery disease of the brain, which could explain the extensive white matter changes and frequent cerebral microbleeds seen in this condition. The vulnerability of the temporal pole might be explained by its enlarged perivascular space due to its unique structure and vascularization by branches of the anterior temporal artery.[13]

One important limitation of the present study is that it was conducted at a single institution and included patients of a single ethnicity. The sensitivity and specificity of temporal lobe hyperintensities for CADASIL diagnosis have been reported as 89% and 86%, respectively, in Caucasian patients, with up to 95% of Caucasian patients with the disease exhibiting these findings [2, 14]. However, the prevalence of temporal lobe involvement in Asian patients with CADASIL was reported to be only 43% to 71% [15–17]. This difference in prevalence might be related to ethnic variations in the mutation profile. Cysteine-sparing mutations in CADASIL, such as Arg75Pro, have been reported in Asia, and such patients have mostly lacked anterior temporal pole involvement [18]. Another limitation is imaging technique heterogeneity, particularly in the present study that encompassed a long time period. However, we confirmed that the percentages of patients scanned with 1.5-T or 3.0-T MRI did not differ between the DM1 and CADASIL groups. In addition, visual rating scales are particularly beneficial in studies in which different imaging devices with varying image qualities are employed [19]. The other limitation is possible selection bias for patients with DM1. Only a relatively small number of patients with DM1 (29/149 [19.5%]) met the inclusion criteria for investigation in this study. Although we showed that the clinical findings were similar among those who did or did not undergo MRI (S1 Table), we cannot rule out selection bias in this group.

Despite these limitations, our investigation has certain strengths. To the best of our knowledge, it is the first to systematically address the similarities and differences of brain MRI in patients with DM1 or CADASIL. We determined a composite ARWMC score cutoff value with high sensitivity and specificity to differentiate the MRI findings in CADASIL from those in DM1. Secondly, our study provides clinicians with valuable information when evaluating a patient found to have temporal pole hyperintensities on MRI. Although DM1 and CADASIL differ in terms of pathogenesis, they share similar clinical and radiologic findings that might lead to a mistaken diagnosis. Furthermore, while assessing patients possessing a very mild DM1 phenotype without major clinical clues to the diagnosis or those with CADASIL presenting with white matter abnormalities on brain MRI but only subtle clinical symptoms, the brain MRI can play an important role in correctly identifying the disease.

In conclusion, anterior temporal lobe involvement in DM1 is not infrequent compared with CADASIL. However, because brain MRI in patients with DM1 lacks other distinctive features seen in CADASIL, imaging might assist in differentiating these two conditions.

## Supporting information

S1 Table Clinical characteristics of patients with DM1 who did or did not undergo brain MRI. (DOCX)

S1 Dataset Study dataset. (XLSX)
