# Supplementary material for: Comparison of brain magnetic resonance imaging between myotonic dystrophy type 1 and cerebral autosomal dominant arteriopathy with subcortical infarcts and leukoencephalopathy
Source: PLoS One. 2018 Dec 6;13(12):e0208620. doi: 10.1371/journal.pone.0208620 (PMC6283577; doi:10.1371/journal.pone.0208620)
Supplement: S1 Table — (DOCX) [file pone.0208620.s001.docx]

**S1 Table. Clinical characteristics of patients with DM1 who did or did not undergo brain MRI.**

|  | With MRI (n=29) | Without MRI (n=120) | *P*-value |
| --- | --- | --- | --- |
| Age (years) | 48.9 ± 14.1 | 46.4 ± 11.6 | 0.442 |
| Male | 17 (58.6) | 53 (44.2) | 0.162 |
| Hypertension | 2 (6.9) | 4 (3.3) | 0.331 |
| Diabetes mellitus | 5 (17.2) | 12 (10.0) | 0.327 |
| Smoking | 5 (17.2) | 10 (8.3) | 0.172 |
| Abnormal ECG^a^ | 17/24 (29.2) | 26/74 (35.1) | 0.591 |
| CTG repeats | 315.5 ± 175.4 | 346.8 ± 200.1 | 0.318 |

Values are presented as mean ± standard deviation or n (%).

^a^Abnormal ECG included any degree atrioventricular block and arrhythmias.

DM1, myotonic dystrophy type 1; ECG, electrocardiography; MRI, magnetic resonance imaging.
